# Smartphone-based stent tracking application for prevention of forgotten ureteral double-J stents: a prospective study

**DOI:** 10.1590/S1677-5538.IBJU.2018.0707

**Published:** 2019-04-01

**Authors:** Volkan Ulker, Hasan Anil Atalay, Ozgur Cakmak, Cem Yucel, Orcun Celik, Zafer Kozacioglu

**Affiliations:** 1 Department of Urology, Health Sciences University, Tepecik Training and Research Hospital, Izmir, Turkey;; 2 Department of Urology, Health Sciences University, Okmeydani Training and Research Hospital, Istanbul, Turkey

**Keywords:** Ureteroscopy, Patient Safety, Urolithiasis

## Abstract

**Purpose:**

Retained or forgotten ureteral stents (FUS) have a potential to cause significant morbidity as well as medico-legal issues and increased cost. We aimed to evaluate the efficacy and usefulness of smartphone-based Ureteral Stent Tracker (UST) application and compare the results with basic appointment card system to prevent FUS, prospectively.

**Materials and Methods:**

A total of 90 patients who underwent ureteroscopic stone treatment procedure with indwelling DJ stents were equally distributed into two groups. In group-1, patients were followed using UST application. In group-2, only appointment cards were given to the patients. Two groups were compared in terms of stent overdue times and complete lost to follow up rates.

**Results:**

Forty-four patients in group-1 and 43 patients in group-2 completed the study. Among patients, 22.7% in group-1 and 27.9% in group-2 did not return for the stent removal on the scheduled day. In group-1, these patients were identified using the UST and called for the stent removal on the same day. After 6 weeks of maximal waiting period, mean overdue times in group-1 and group-2 were 3.5 days and 20 days, respectively (p = 0.001). In group-2, 3 patients (6.9%) were lost to follow up, while in group-1, it was none (p = 0.001).

**Conclusions:**

We found that the patients who were followed by the smartphone-based UST application has less overdue times and lost to follow up cases compared to the basic appointment card system. The UST application easily follows patients with indwelling ureteral stents and can identify patients when overdue.

## INTRODUCTION

Ureteral double-J (DJ) stent placement is one the most common procedures in daily urological practice. The DJ stent placement is indicated in the treatment of urolithiasis, to relieve benign or malign obstruction, to promote ureteral healing and manage urinary leak (1). Most DJ stents are inserted for temporary purposes and need to be removed on maximal safe life depending on their production material. However, approximately 12% of all ureteral DJ stents are retained or forgotten (2). These forgotten ureteral stents (FUS) may lead to infection, migration, encrustation and fragmentation (3, 4). El-Faqih et al. reported that encrustation occurred in 9.2% of the stents under 6 weeks, 47.5% between 6-12 weeks and 76.3% after 12 weeks in removed DJ stents which were placed for urolithiasis (5). Furthermore, more serious complications such as sepsis, renal failure or even mortality have been reported with FUS (6). Besides the additional cost and medico-legal problems, management and removal of encrusted and infected FUS may require combined endourologic procedures and may represent a challenge for urologists (7). The attending surgeon is responsible for both monitoring the patient and safe removal of the stent. Therefore, tracking of patients with indwelling DJ stents and stent removal on a planned time is quite important to avoid increased morbidity and healthcare costs.

In order to prevent FUS, different stent tracking and registry systems have been developed including paper card registry (8), electronic patient registry (2, 4) and computer based e-mail (9) or short-message-service (SMS) reminders (10). However, these systems presented a solution only for a single institute. They required infrastructure with extra cost and secretarial entry to the system.

Today, smartphones are an integral part of our daily lives. For tracking patients with indwelling DJ stents, a cloud based smartphone application, Ureteral Stent Tracker™ (UST) was developed (11). In a prospective study, we aimed to evaluate the efficacy and usefulness of UST application and compare the results with basic appoinment card system to prevent FUS.

## MATERIALS AND METHODS

After obtaining the approval of institutional ethics committee, a prospective non-randomized study was created. Patients between the age of 18 and 80 who underwent ureteroscopic laser lithotripsy for urinary stone disease and followed by DJ stent placement in our clinic were included the study. Ureteral DJ stent indications other than ureteroscopic stone surgery, patients with long-term or metallic stents for malignancy and patients with language problems were excluded to create a more homogenous patient group. Between April 2018 and July 2018, 90 of 104 patients were found eligible. In all patients, 4.8 French (F), 24-28 cm Percuflex (Boston Scientific, Marlborough, MA, USA) DJ stent was used. The indwelling time for stents was described as 2 weeks. Bilateral ureteral DJ stents were counted as 1-stent care plan if inserted simultanesously. Patients received only non-steroidal analgesics on-demand.

Patients were equally distributed into two groups consecutively based on their date of operation. In group 1, 45 patients with indwelling DJ stents were recorded to the UST application running on an iPhone 6S smartphone (Apple Inc., Cupertino, CA, USA) in addition to the appointment card for stent removal. Every day, the UST visual dashboard was reviewed by the follower to check patients with indwelling stents. In this group, patients who did not return for stent removal on the scheduled date were contacted by phone and their appointments were reminded by the follower. Patients who were unreachable were called again, once a day for 2 days. In group 2, only an appointment card was given to the patients and they were asked to return to the hospital on a scheduled date for stent removal. In both groups, patients were also verbally informed about the indwelling stent. Since there is no stent registry, this is the standart procedure in our institution. Patients in group 2 were not called to remind their appointments for stent removal. For ethical reasons, the maximal waiting period for the patients who did not return to the hospital for stent removal was limited to 6 weeks. The patients were contacted by phone in a protocol described above and invited to the hospital for stent removal if they exceeded the 6 weeks maximal waiting period. Two groups were compared in terms of stent overdue times and lost to follow-up rates.

**Figure 1:**
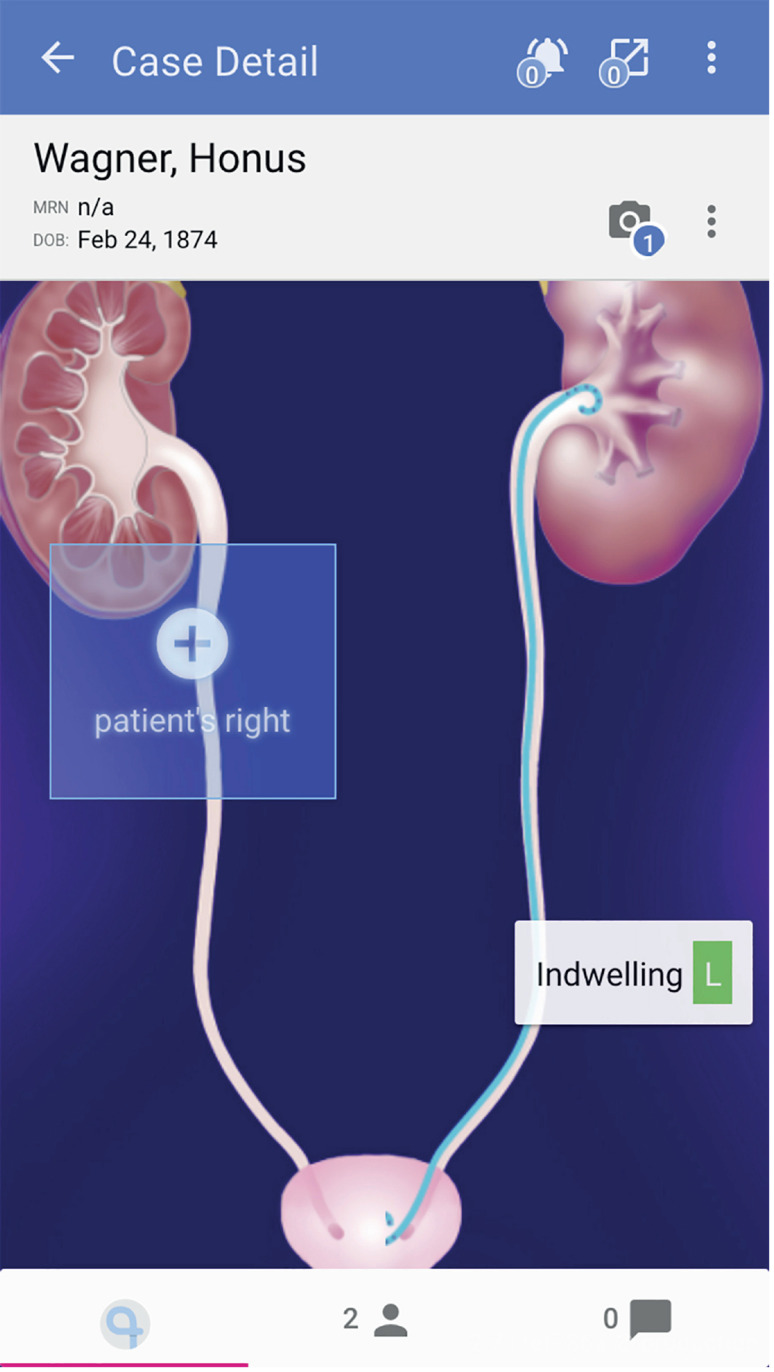
A view of ureteral stent care plan of patient (Images© Copyright Visible Health, Inc. Created for demonstration).

**Figure 2:**
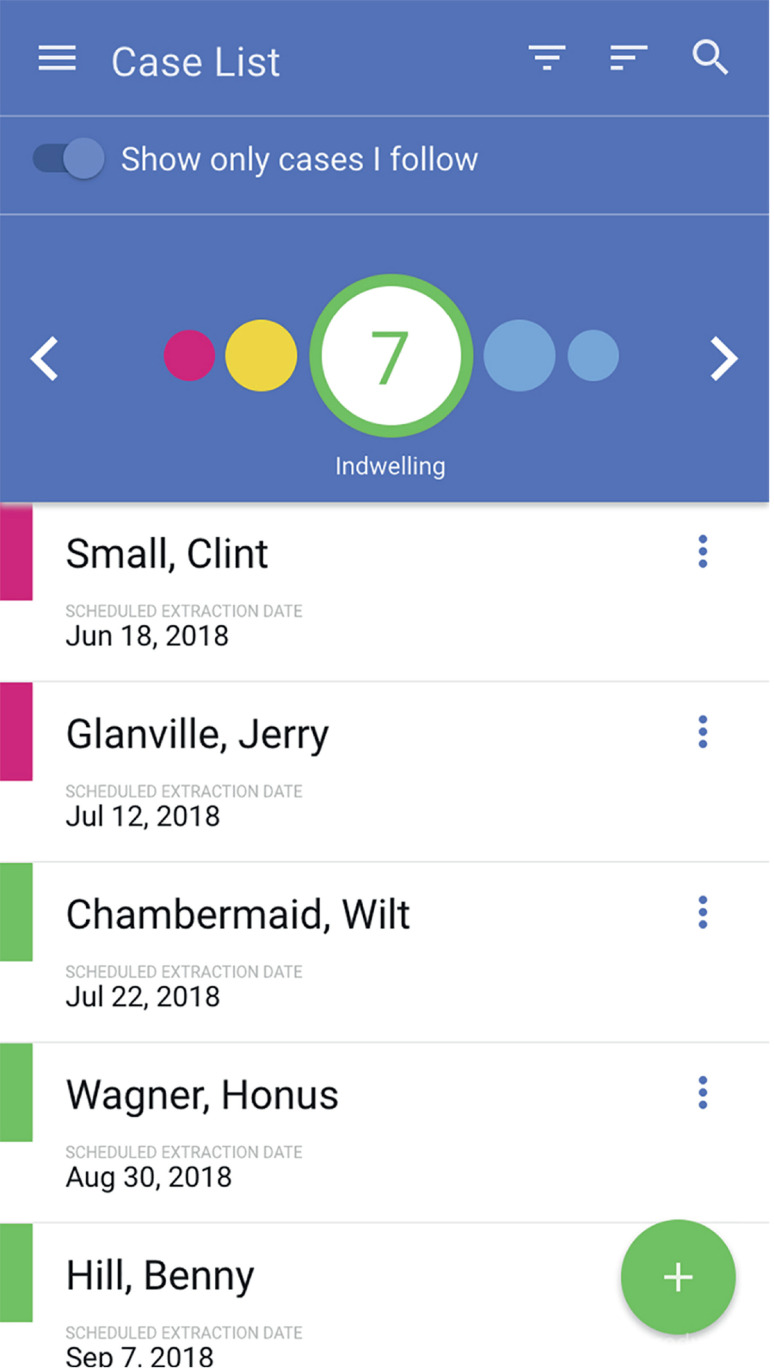
A view of the UST dashboard showing overdue, incomplete, indwelling, within 2 weeks and extracted cases (Images© Copyright Visible Health, Inc. Created for demonstration).

UST* was developed by Visible Health Inc. (Austin, TX, USA) in partnership with Boston Scientific and can be downloaded from Apple Store or Google Play. However, its use is limited to physicians who are registered and pre-authorized by Boston Scientific. The UST is a password-protected, encripted, cloud-based and HIPAA compliant application for smartphones which a web browser interface also exists. The administator and privacy officer of Visible Health team have access to patient data for maintenance and support. After registering a patient with name and medical record number, and scanning the stent product barcode, the responsible physician can create a care plan and schedule a stent extraction date (Figure-1). Creating a new case is at the POC and real-time. The application allows users to review overdue, incomplete, and indwelling lists within 2 weeks, and extracts stent patient groups (Figure-2). Once a default follower is set up, additional followers can be added to the care plan by the primary follower. Each created profile shows patient information including e-mail address and phone number, stent insertion date, laterality, scheduled stent removal date, and extraction date if the stent was already removed. It is also possible to send an e-mail reminder and attach patient information guide about the DJ stent to patients (only in English yet) if the administrator turns on this hidden feature. After the set up, UST sends daily or weekly reminder e-mails to the follower showing the list of patient groups described above.

The statistics were presented as mean ± standard deviation (SD). Distribution of variables was assessed with the Kolmogorov-Smirnov test. The Student's t test and Mann-Whitney u test were used to compare independent quantitative data. Chi-square test was used to compare qualitative data. The power of the study was calculated using the G* Power program (University of Dusseldorf, Dusseldorf, Germany), an effect size convention of 0.8 for the two-tailed t-test with an alpha error protection of 0.05. Statistical significance was assessed with two-tailed tests, and p was considered to be statistically significant when < 0.05. Statistical tests were performed using SPSS 22.0 (IBM Corp., Armonk, NY, USA).

## RESULTS

Eighty-seven of 90 patients with 87 ureteral stent care plans (44 patients in group 1 and 43 patients in group 2) completed the study. The patient's demographics are shown in Table-1. There were bilateral DJ stents in 2 patients in group 1, and none in group 2. One patient in group 1 and two patients in group 2 were exluded because their stents were removed before the scheduled date due to severe discomfort.

**Table 1:** Patients’ demographics.

		Min-Max	Median	Mean±s.d/n-%		
**Age**		20.0-80.0	48.0	48.6	±	14.5
	Male			54		62.0%
**Sex**					
	Female			33		38.0%
	Primary			27		31.0%
**Education level**					
	High			36		41.4%
	University			24		27.6%
	Left			43		49.4%
**Stent side**					
	Right			42		48.2%
	Bilateral			2		2.4%

There was no statistical difference between two groups in terms of age, sex, educational level and laterality (Table-2). In group 1, which was tracked with the UST application, 10 / 44 (22.7%) patients missed their appointments for stent removal and returned with a mean 2.5 ± 0.9 days after a phone call by the follower. The reasons for the delay in this group were social issues in 3, and health problems in 7 cases. Similarly, in group 2, 12 / 43 (27.9%) patients missed their appoinments. On the other hand, 9 of 12 patients who missed their appointments in group 2 returned for stent removal within 6 weeks period with a mean 16.3 ± 5.0 days overdue.

**Table 2:** Results and comparison of the two groups.

		Group 1				Group 2				*p*	
		Mean±s.d/n-%			Median	Mean±s.d/n-%			Median		
**Age**		50.0	±	15.0	48.5	47.2	±	14.0	46.0	0.430	t
	Male	25		73.5%		19		57.6%		0.169	χ2
**Sex**											
	Female	9		26.5%		14		42.4%			
	Primary	9		26.5%		10		30.3%			
**Education level**											
	High	15		44.1%		14		42.4%		0.439	χ2
	University	10		29.4%		9		27.3%			
	Left	17		50.0%		16		48.5%		0.901	χ2
**Side**											
	Right	17		50.0%		17		51.5%	
**Overdue** * **(day)**		2.5	±	0.9	1.9	16.3	±	5.0	12.2	0.001	m

t = t test;

m = Mann-whitney u test;

χ2 = Chi-square test;

* = patients not returning for stent removal

The delay reasons of these 9 patients were social issues in 7 and health problems in 2. The remaining 3 patients (6.9%) were considered as lost to follow-up and contacted by phone and invited for stent removal since they had exceeded 6-week-maximal-waiting-period for the study. In these 3 cases, forgetting the existence of the stent was the main reason for failing to return for stent removal. All stents were removed in both groups. Among patients who did not return for stent removal, statistical evaluation revealed that patients in group 1 had significantly less overdue times (p = 0.001) and lost to follow-up cases (p = 0.001) compared to group 2.

## DISCUSSION

With developments in endourology and minimal invasive procedures, the numbers of indwelling DJ stents have increased. The high volume of ureteral DJ stents increase the number of retained or FUS. The reasons of FUS are not clear. Divakaruni et al. have tried to identify the risk factors for FUS (2). They concluded that males were 2.5 times more likely to have FUS than females, and patients without insurance were nearly 6 times more likely to have FUS compared to insured patients. Employment status, educational level, and ability to speak English were not in association with FUS in this study.

Despite progress in stent and biomaterial technology, FUS is still associated with significant morbidity. The most commonly used DJ stents are polyurethane and have an average indwelling time ranging from 3 to 6 months. It is clear that there is a correlation between the ureteral stent indwelling time and biofilm formation and encrustation (5, 12). Kawahara et al. investigated stent encrustation and morbidity related to indwelling time (3). In a total of 330 stents, they found that the encrustation rate was 26.8% in less than 6 weeks, 56.9% in 6 to 12 weeks, and 75.9% in more than 12 weeks. Monga et al. reported a series of FUS left in situ for a mean of 22.7 months, and 68% were calcified, 45% were fragmented, and 14% were calcified and fragmented, respectively (7). These infected and encrusted FUS may lead to complications ranging from simple urinary system infection to septic shock and even death (6). The negative effect on glomerular filtration rate (13) and renal failure has also been reported with FUS (14). Furthermore, management of infected and encrusted FUS can be a challenge, especially in solitary kidneys. Removal of a highly encrusted FUS may require extracorporeal shockwave lithotripsy (ESWL) or endourologic procedures including cyctolithopaxy, ureterorenoscopy (URS) and percutaneous nephrolithotomy (PCNL), or combination of these procedures (15–17). Open or laparoscopic surgery is rarely needed to remove an encrusted DJ stent (18).

Another important issue on FUS is the medicolegal consequences. Although patients are well informed with their in situ DJ stents, any complication related to FUS will be the responsibiliy of the attending surgeon. Duty et al. reviewed closed malpractice claims in New York State and revealed that in a total of 585 claims against the urologist, 4 claims were due to retained DJ stents (19). Failure to arrange proper follow-up resulting in retained DJ stents was alleged in 27% of dismissed cases. In a similar study, Osman and Collins reviewed data on urological litigation within the UK National Health Service (NHS) between the years 1995 and 2009 (20). The largest category for dissatisfaction with care was postoperative-related claims, and within these, forgotten ureteral stents were 23 cases in a total of 168 claims.

Besides the identification of the risk factors, the development of an effective method for the prevention of FUS is quite important. In order to prevent FUS, a number of patient registry system has been used. Tang et al. tested card registry system in this manner (8). They retrospectively reviewed their card registry for a 5-year-period and reported a 94.1% success rate in registering the patients. However, the registry of 5.9% has been missed in operating theatre due to human error. Additionally, 25% of the patients had no records of stent extraction. The card registry system was concluded to be ineffective. Similarly, Thomas et al. evaluated their ureteral stent logbook system and reported that 22.4% of the patients were unaccounted, largely as the stent removal was not documented by the surgeon (21). These written sytems seem to be ineffective since they need teamwork for double-checking and paperwork. Also, remote access to the registry is not possible.

Electronic patient registries and computer-based tracking systems were also described to prevent FUS. In 1996, McCahy and Ramsden introduced a computer database which was reviewed by administrative staff monthly to capture patients with overdue stents (22). They demonstrated a reduction in the number of overdue DJ stents from 3.6% to 1.1%. Ather et al. also reported a computer database that reduced the rate of overdue stents from 12.5% to 1.2% (23). Even though these systems were better than the card registry systems, they still required manual data entry and manual review. In 2007, Lynch at al. reported an electronic stent register which was integrated to the network and EMR system of the hospital (9). After stent insertion and entry of maximal stent life, this system sent a notice by e-mail to clinical staff if a stent became overdue for removal. They reported that 13% of the stents were not captured even after barcode implementation. Although this electronic registry system was more succesful than paper-based systems, it needed specific programs and access to the network of the center. To eliminate the uncaptured stent problem, Baumgarten at al. described a billing-based system where the entry of the ICD-9 ureteral catheterization code with CPT and HCPCS codes to the system automatically recorded the patient to the stent registry and sent an HIPAA compliant reminder letter to the patient (24). However, this system has incorrectly captured many patients with non-ureteral stents. Some other computer-based stent registries sending SMS reminders instead of e-mails have also been described (10, 25).

All the mentioned systems have some limitations. In the card-based systems, human factor is a main limitation. Manual entry and review are needed in addition to a lot of paperwork. Loss of patient records and archiving are also problems. Although electronic registries and computer-based systems have some advantages over card-based systems, they need specific programs, access to the hospital network, and extra costs. Moreover, these systems are only available to their developer-institutions which are usually high volume centers.

With the advances in cellular phone technology, medical applications running on smartphones have started to develop rapidly. In 2017, Molina et al. reported their retrospective study using the UST (11). In this study, 77% of stents were removed on time while 9% were overdue. However, remaining 14% were scheduled to be removed by the time of analysis. Only 1 out of 194 patients were lost to follow-up. After this study, Ziemba et al. reported that 3 out of 115 patients (3%) who did not return for their scheduled stent removal could be identified only through the UST application (26). In our study, using UST, we could easily identify patients who failed to return for stent removal. In addition, these patients had less overdue times compared to patients with appoinment cards. However, all patients with overdue times can not be cathegorized as FUS. The majority of patients with overdue times returned for removal in a safe period. On the other hand, 3 patients in the appoinment card group who did not return after 6 weeks of maximal waiting period can be considered as FUS candidates.

Despite the advantages of UST over previously described systems, there are some weak points. First, the UST is not integrated to the institutional EMR. Secondly, there is no other alternative for the registry of stent removal by an urologist other than the follower. Our study has some limitations; the number of patients is not high, and we had to limit the maximal waiting period to 6 weeks due to ethical reasons. Additionally, closer follow-up of the patients in group 1 and no phone calls for group 2 until the maximal waiting period was reached might bring about a bias and better outcomes for group 1.

## CONCLUSIONS

We found that the patients with indwelling DJ stents who were followed by the smartphone-based UST application has significantly less overdue times and lost to follow-up cases. The UST is secure and easy to use POC application, and it allows urologists to check their patients out of the institute since it is cloud-based. Compared to basic appointment card system, the UST allows effectivelly tracking of the patients with indwelling stents and identifying them more quickly if they fail to return for stent removal.

## ABBREVATIONS

HIPAA: = Health Insurance Portability and Accountability Act of 1996
EMR: = Electronic Medical Record
ICD-9: = International Classifications of Disease, 9th revision
CPT: = Current Procedural Terminology
HCPCS: = Healthcare Common Procedure Coding System
OR: = Operating Room
